# Difference in gene duplicability may explain the difference in overall structure of protein-protein interaction networks among eukaryotes

**DOI:** 10.1186/1471-2148-10-358

**Published:** 2010-11-18

**Authors:** Takeshi Hase, Yoshihito Niimura, Hiroshi Tanaka

**Affiliations:** 1 Department of Bioinformatics, Medical Research Institute, Tokyo Medical and Dental University, Yushima, Bunkyo-ku, Tokyo 113-8510, Japan; 2 Department of Bioinformatics, Graduate School of Biomedical Science, Tokyo Medical and Dental University, Yushima, Bunkyo-ku, Tokyo 113-8510, Japan

## Abstract

**Background:**

A protein-protein interaction network (PIN) was suggested to be a disassortative network, in which interactions between high- and low-degree nodes are favored while hub-hub interactions are suppressed. It was postulated that a disassortative structure minimizes unfavorable cross-talks between different hub-centric functional modules and was positively selected in evolution. However, by re-examining yeast PIN data, several researchers reported that the disassortative structure observed in a PIN might be an experimental artifact. Therefore, the existence of a disassortative structure and its possible evolutionary mechanism remains unclear.

**Results:**

In this study, we investigated PINs from the yeast, worm, fly, human, and malaria parasite including four different yeast PIN datasets. The analyses showed that the yeast, worm, fly, and human PINs are disassortative while the malaria parasite PIN is not. By conducting simulation studies on the basis of a duplication-divergence model, we demonstrated that a preferential duplication of low- and high-degree nodes can generate disassortative and non-disassortative networks, respectively. From this observation, we hypothesized that the difference in degree dependence on gene duplications accounts for the difference in assortativity of PINs among species. Comparison of 55 proteomes in eukaryotes revealed that genes with lower degrees showed higher gene duplicabilities in the yeast, worm, and fly, while high-degree genes tend to have high duplicabilities in the malaria parasite, supporting the above hypothesis.

**Conclusions:**

These results suggest that disassortative structures observed in PINs are merely a byproduct of preferential duplications of low-degree genes, which might be caused by an organism's living environment.

## Background

Large-scale data of protein-protein interactions have become available from several organisms, including *Saccharomyces cerevisiae *(yeast; [1-4]), *Caenorhabditis elegans *(worm; [5]), *Drosophila melanogaster *(fly; [6]), *Homo sapiens *(human; [7,8]), and *Plasmodium falciparum *(malaria parasite; [9]). In a protein-protein interaction network (PIN), a protein and an interaction between two proteins are represented as a node and a link, respectively. The number of links connected to a node is called a degree. The degree distribution *P*(*k*) represents the fraction of *k*-degree nodes in a network and characterizes the structure of a network. It is well known that various biological, technological, and social networks are scale-free networks, in which *P*(*k*) follows a power law, *i.e.*, *P*(*k*) ~ *k^-γ ^*[10-12]. In a scale-free network, therefore, most of the nodes have low degrees, but a small number of high-degree nodes (hubs) also exist. In the case of PINs, *P*(*k*) better fits a power law with an exponential cut-off, *i.e.*, $P\left(k\right)\sim {\left(k_{0}+k\right)}^{-}{}^{\gamma }{\text{e}}^{-k/k_{\text{c}}}$[13,14].

A correlation between degrees of two nodes connected by a link is another feature characteristic of a network architecture. A simple way to see the degree correlation is to consider the Pearson correlation coefficient *r *of the degrees at both ends of a link [12,15,16]. A network is called as assortative when *r *> 0, while it is disassortative when *r *< 0. In an assortative network, hubs are preferentially connected to other hubs, whereas in a disassortative network, hubs tend to attach to low-degree nodes. It was reported that social networks such as coauthorships of scientific papers or film actor collaborations are assortative, whereas technological and biological networks including Internet, food web, neural network, and PIN are disassortative [16].

Assortativity of a network can also be evaluated by <*K*_nn_(*k*)>, the mean degree among the neighbors of all *k*-degree nodes ("nn" in <*K*_nn_(*k*)> represents "nearest neighbors"; [12,14,17,18]). In assortative and disassortative networks, <*K*_nn_(*k*)> follows an increasing and decreasing functions of *k*, respectively. If there are no degree correlations, <*K*_nn_(*k*)> is independent of *k*, <*K*_nn_(*k*)> = <*k*^2^>/<*k>*[12]. Several studies reported that the yeast PIN is a disassortative network showing <*K*_nn_(*k*)> ~ *k*^-*ν *^[12,14,17], where *ν *represents the extent of disassortative structure. In the yeast PIN, therefore, links between a hub and a low-degree node are favored, but those between hubs are suppressed. From this observation, Maslov and Sneppen [17] suggested a picture that, in the yeast PIN, a hub forms a functional module of the cell together with many low-degree neighbors. They hypothesized that the suppression of interactions between hubs minimizes unfavorable cross-talks between different functional modules and increases the robustness of a network against perturbations. Therefore, it is postulated that the disassortative structure in the yeast PIN has been favored by natural selection. Note that, if this hypothesis is true, a disassortative structure should be a general feature that is commonly observed among PINs in any organisms.

To understand the evolutionary mechanisms shaping PIN architectures, several network growth models have been proposed. Many of them are based on gene duplication and divergence, in which a randomly selected node is duplicated to generate a new node having the same links as the original node, and some links are added or eliminated in a divergence process [19-23]. We have recently proposed a non-uniform heterodimerization (NHD) model [14]. In this model, a new link is preferentially attached between two duplicated nodes to create a cross-interaction when they share many common neighbors. We showed that this model can the best reproduce structural features of the yeast PIN, including scale-freeness, a small number of cross-interactions, and a skewed distribution of triangles composed of three nodes and three links. However, this model as well as other duplication-divergence models [21,22] failed to explain the presence of a disassortative structure in the yeast PIN. Simulation studies showed that these models could generate a decreasing function of <*K*_nn_(*k*)>, yet the value of *ν *(0.18) in <*K*_nn_(*k*)> ~ *k*^-*ν *^is much smaller than the actual value (0.47; see Tables 1 and 2). Therefore, the origin of a disassortative structure still remains unexplained. We should again note that most of these simulation studies were carried out by using the yeast PIN only, because it is currently the best characterized.

It is well-known that large-scale PIN data contain many false positive interactions [24]. Maslov and Sneppen [17] used a dataset obtained by high-throughput yeast two-hybrid (Y2H) screens [2] to show suppression of interactions between high-degree nodes. Aloy and Russell [25], however, argued that the observed suppression of hub-hub interactions is probably an artifact caused by a systematic error in the Y2H data due to prey-bait asymmetry (see also Maslov and Sneppen [26]). To circumvent the problem of high false positive rates in high-throughput datasets, Batada et al. [27] used only interactions that were independently reported at least twice in different datasets, and they found that hub-hub interactions were not suppressed in the multi-validated yeast PIN data. However, Hakes et al. [28] pointed out that multiple validation introduces another problem: interactions observed at least twice will be biased towards well-studied proteins, such as those from particular cellular environments or highly expressed ones. They showed that assortativity of a PIN drastically changes depending on datasets [28]. A literature-curated yeast PIN dataset [29], which is expected to be reliable because each of the interaction data was derived from small-scale experiments, showed a disassortative structure; however, when they retained only interactions observed twice or three times, it became rather assortative [28]. Therefore, the presence of a disassortative structure in a PIN itself has now become controversial. These studies suggest that a global structure of a PIN has to be investigated by using various datasets obtained from different methods.

The purpose of this paper is to investigate the presence of disassortative structures in PINs and an evolutionary mechanism shaping disassortative structures, if any. For this purpose, we examined eukaryotic PINs from the yeast, worm, fly, human, and malaria parasite. We analyzed four large-scale yeast PIN datasets (MIPS [3]; Yu et al. [4]; Reguly et al. [29]; Batada et al. [30]). The datasets include Batada et al.'s updated version of a multi-validated dataset, Reguly et al.'s comprehensive literature-curated dataset, and MIPS [3], which has been called a "gold standard" of yeast protein interaction dataset generated by manual curations by experts. We also used recently published high-quality protein interaction data by Yu et al. [4], which were obtained by compiling several Y2H datasets. In addition, we examined two independent human PIN datasets (Rual et al. [7]; Stelzl et al. [8]). As a result, we show that the yeast, worm, fly, and human PINs have disassortative structures, while malaria parasite PIN is not disassortative. We then propose a possible evolutionary mechanism causing the difference in assortativity among species.

## Results

**Figure 1:**
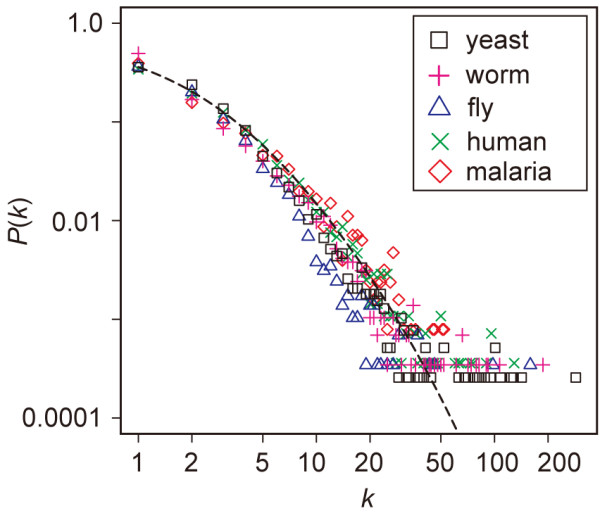
**Degree distribution of PINs in five eukaryote species**. Degree distribution *P*(*k*) in the PINs of yeast (black square), worm (magenta plus), fly (blue triangle), human (green cross), and malaria parasite (red diamond). For yeast and human PINs, *P*(*k*) for MIPS and Rual et al. datasets, respectively, are shown, because they contain the largest numbers of genes among the PINs for each species. The results for the other yeast and human datasets are provided in Additional file 1: Figure S1. A dashed line represents ${\left(k_{0}+k\right)}^{-}{}^{\gamma }{\text{e}}^{-k/k_{\text{c}}}$ with *γ *= 2.7, *k*_0 _= 3.4, and *k*_C _= 50.

In this study, we examined nine PIN datasets from yeast, worm, fly, human, and malaria parasite (Table 1). Although the numbers of nodes and links are quite different among the five species, their degree distributions *P*(*k*) follow nearly the same curve (Figure 1 and additional file 1: Figure S1). All of the PINs examined are scale-free, suggesting that scale-freeness is a general feature of PINs. These observations are consistent with Suthram et al. [31].

**Table 1:** Statistics of the PINs from five eukaryote species

Species	Dataset	Data type	***n***^**a**^	*# *of links	***ν***^**b**^	**<*k*>**^**c**^	***<C>***^**d**^	***r***^**e**^	***<L>***^**f**^	***M***^**g**^
Yeast	MIPS	Manually curated	3,891	7,270	0.47***	3.74	0.066	-0.14***	4.85	0.662
	Yu et al. (2008)	Y2H	1,647	2,518	0.25***	3.06	0.057	-0.11***	5.61	0.739
	Batada et al. (2007)	Multi-Validated	3,801	9,742	0.33***	5.13	0.171	-0.12***	4.69	0.715
	Reguly et al. (2006)	Literature curated	3,224	11,291	0.33***	7.00	0.266	-0.13***	4.22	0.689

Worm	Li et al. (2004)	Y2H	2,898	5,240	0.29***	3.62	0.072	-0.14***	4.95	0.679

Fly	Pacifico et al. (2006)	Y2H	2,477	3,546	0.35***	2.87	0.025	-0.09***	5.93	0.738

Human	Rual et al. (2005)	Y2H, Literature Curated	2,783	6,007	0.26***	4.32	0.072	-0.14***	4.84	0.630
	Stelzl et al. (2005)	Y2H	1,613	3,101	0.27***	3.85	0.006	-0.20***	4.85	0.588

Malaria parasite	LaCount et al. (2005)	Y2H	1,267	2,726	0.02	4.30	0.014	-0.03*	4.26	0.506

a. Number of nodes in a network.

b. The extent of disassortative structure. *** indicates a significantly non-zero value (*P *< 0.001).

c. The mean degree.

d. The mean cluster coefficient. The cluster coefficient of node i is defined as *C*_i _= 2*e*_i_/*k*_i_(*k*_i_-1), where *k*_i _is the degree of node i and *e*_i _is the number of links connecting *k*_i _neighbors of node i to one another [67]. When *k*_i _is one, *C*_i _is defined to be zero. *C*_i _is equal to one when all neighbors of node i are fully connected to one another, while *C*_i _is zero when none of the neighbors are connected to one another.

e. The Pearson correlation coefficient between degrees of two nodes connected to each other. *, *P *< 0.05; **, *P *< 0.01; ***, *P *< 0.001.

f. The mean shortest path length, which is defined as the mean of the shortest path length between all pairs of nodes in a network [14].

g. The modularity. See Methods.

**Figure 2:**
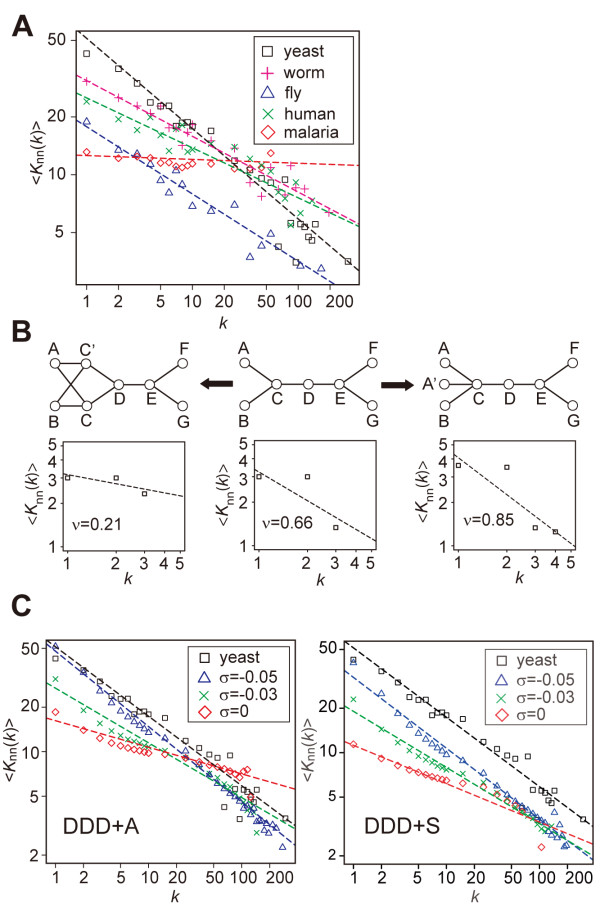
**Difference in assortativity among eukaryote PINs**. **(*A*) **<*K*_nn_(*k*)>, the mean of the degrees among the neighbors of *k*-degree nodes, in the PINs of yeast (black square), worm (magenta plus), fly (blue triangle), human (green cross), and malaria parasite (red diamond). For yeast and human PINs, <*K*_nn_(*k*)> for MIPS and Rual et al. datasets, respectively, are shown, and the results for the other yeast and human datasets are provided in Additional file 2: Figure S2. Dashed lines in black, magenta, blue, green, and red represent *k*^-0.47^, *k*^-0.29^, *k*^-0.35^, *k*^-0.26^, and *k*^-0.02^, respectively. **(*B*) **Duplication of a node changes the value of *ν *in <*K*_nn_(*k*)> ~ *k*^-*ν*^. A diagram below each network indicates the distribution of <*K*_nn_(*k*)> and the value of *ν*. **(*C*) **The distribution of <*K*_nn_(*k*)> in the networks generated by the DDD model with the asymmetric divergence (DDD+A; left) and the symmetric divergence (DDD+S; right). Blue diamonds, green crosses, and red diamonds indicate the results with *σ *= -0.05 (-0.05), -0.03 (-0.03), and 0 (0), respectively, for DDD+A (DDD+S). These results were obtained by taking the mean among 100 networks generated by simulations. Black squares indicate <*K*_nn_(*k*)> in the yeast PIN for MIPS. Dashed lines in black, blue, green, and red represent *k*^-0.47 ^(*k*^-0.47^), *k*^-0.51 ^(*k*^-0.48^), *k*^-0.37 ^(*k*^-0.38^), and *k*^-0.18 ^(*k*^-0.26^), respectively, for DDD+A (DDD+S).

On the other hand, a disassortative structure was not commonly observed among PINs. Although <*K*_nn_(*k*)> for the yeast, worm, fly, or human PIN is a decreasing function following *k*^-*ν*^, the malaria parasite PIN is not disassortative (Figure 2A and additional file 2: Figure S2). Note that all of the four yeast PIN datasets showed a disassortative structure regardless of the controversy on the presence of hub-hub suppression (see additional file 2: Figure S2; see Discussion). The values of *ν *for the eight PINs in yeast, worm, fly, and human examined are significantly non-zero (*P *< 3×10^-4^), while the value of *ν *for the malaria parasite PIN is not significantly different from zero (*P *~ 0.27). The difference in *ν *between the malaria parasite PIN and each of the other eight PINs is also significant (*P *< 1×10^-3^; analysis of covariance). In agreement with these observations, the correlation coefficient *r *between degrees of connected nodes in the yeast, worm, fly, or human PIN is negative, while that in the malaria parasite PIN is nearly zero (Table 1).

We next examined a possible evolutionary scenario generating the difference in assortativity of PINs among species on the basis of a duplication-divergence model. Figure 2B (middle) illustrates a simple network containing a low-degree node (e.g., A) and a high-degree node (e.g., C) that are connected to each other. In a duplication process, a randomly selected node is duplicated to generate a new node having the same links as the original node, followed by a divergence process in which some links are eliminated. If a low-degree node A is duplicated to generate a new node A' (Figure 2B, right), the value of *ν *in a network increases, because a degree of a node (C) connected to a low-degree node increases. On the other hand, duplication of a high-degree node (C) causes the value of *ν *to decrease, because a degree of a node (A) connected to a high-degree node increases (Figure 2B, left). Therefore, we can hypothesize that duplications of low- and high-degree nodes in a disassortative network have an effect to make the value of *ν *larger and smaller, respectively.

To examine this issue in more detail, we developed a new duplication-divergence model named the degree-dependent duplication (DDD) model by modifying the NHD model that we proposed previously [14]. In the DDD model, a duplication of a node occurs depending on its degree. In a duplication process, a randomly selected node is duplicated with a probability proportional to 1 + *σk*, where *k *is the degree of the node, and *σ *is a parameter determining the duplicability of the node (see Methods for details).

**Figure 3:**
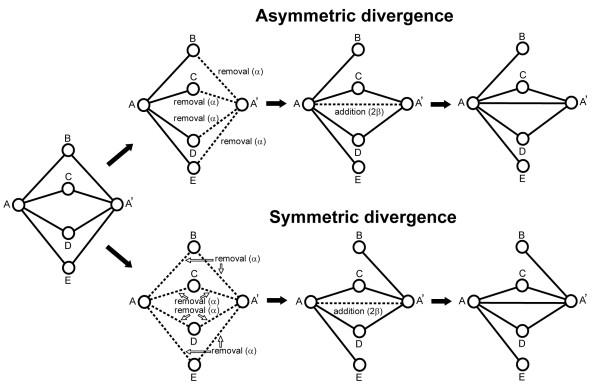
**Degree-dependent duplication (DDD) model**. In the DDD model, the probability of a duplication of a node is dependent on the degree of the node. In the network at the left, node A is duplicated to generate node A' with the probability of (1 + 4*σ*)/1,000, because the degree of node A is four (see Methods). In the asymmetric divergence, each of the links to node A' is removed with a uniform probability *α *in the divergence process (top, second column). In the symmetric divergence, one of the two duplicated links (e.g. either A-B link or A'-B link) to each node connecting to A and A' (nodes B-E) is eliminated with a probability *α *(bottom, second column). A new link between nodes A and A' is attached with the probability proportional to the number of common neighbors (*n*_N_) shared by these nodes (third column). In this case, the probability is 2*β*, because these nodes share two common neighbors (nodes C and D).

As for a divergence process, we examined two different models, the asymmetric divergence and the symmetric divergence (Figure 3). In the former, the removal of links occurs in only one of the duplicated nodes, while in the latter, links are lost from both of the duplicates with an equal probability. In this study, we conducted simulations using four different models: NHD with the asymmetric and symmetric divergence, which is referred to as NHD+A and NHD+S, respectively, and DDD with the asymmetric and symmetric divergence (DDD+A and DDD+S, respectively) (Table 2).

**Table 2:** Statistics of the networks generated by the NHD and DDD models

Species	Model	***n***^**a**^	***σ***^**b**^	*ν*	***α***^**b**^	***β***^**b**^	**<*k*>**^**a**^	***<C>***^**a**^	***<L>***^**a**^	***M***^**a**^
Yeast	NHD+A	3,891	0	0.18	0.75	0.028	3.74 (0.07)	0.066 (0.006)	6.23 (0.12)	0.837 (0.008)
	DDD+A	3,891	-0.05	0.51	0.50	0.019	3.73 (0.11)	0.065 (0.006)	5.63 (0.12)	0.853 (0.008)
	NHD+S	3,891	0	0.26	0.62	0.042	3.76 (0.13)	0.069 (0.005)	8.13 (0.36)	0.866 (0.012)
	DDD+S	3,891	-0.05	0.48	0.49	0.026	3.71 (0.15)	0.066 (0.007)	6.27 (0.16)	0.862 (0.010)

Worm	NHD+A	2,898	0	0.18	0.76	0.032	3.62 (0.08)	0.071 (0.006)	6.10 (0.11)	0.831 (0.009)
	DDD+A	2,898	-0.02	0.31	0.70	0.029	3.58 (0.09)	0.075 (0.008)	6.00 (0.13)	0.841 (0.009)
	NHD+S	2,898	0	0.25	0.63	0.048	3.61 (0.11)	0.073 (0.004)	7.93 (0.36)	0.889 (0.011)
	DDD+S	2,898	-0.01	0.28	0.62	0.046	3.70 (0.10)	0.073 (0.006)	7.76 (0.33)	0.880 (0.011)

Fly	NHD+A	2,477	0	0.18	0.86	0.016	2.84 (0.04)	0.026 (0.004)	6.41 (0.11)	0.860 (0.006)
	DDD+A	2,477	-0.03	0.34	0.80	0.015	2.85 (0.05)	0.027 (0.004)	6.34 (0.12)	0.865 (0.006)
	NHD+S	2,477	0	0.25	0.67	0.020	2.90 (0.08)	0.025 (0.004)	9.11 (0.45)	0.885 (0.010)
	DDD+S	2,477	-0.02	0.34	0.65	0.018	2.91 (0.09)	0.024 (0.004)	8.63 (0.41)	0.883 (0.011)

Human	NHD+A	2,783	0	0.17	0.68	0.025	4.28 (0.13)	0.070 (0.008)	5.79 (0.12)	0.802 (0.011)
	DDD+A	2,783	-0.02	0.28	0.62	0.024	4.25 (0.13)	0.070 (0.007)	5.65 (0.12)	0.814 (0.011)
	NHD+S	2,783	0	0.25	0.58	0.035	4.38 (0.13)	0.070 (0.007)	6.93 (0.20)	0.826 (0.012)
	DDD+S	2,783	0	0.25	0.58	0.035	4.38 (0.13)	0.070 (0.007)	6.93 (0.20)	0.826 (0.012)

Malaria parasite	NHD+A	1,267	0	0.22	0.64	0.004	4.29 (0.15)	0.015 (0.004)	5.28 (0.10)	0.752 (0.014)
	DDD+A	1,267	1.00	-0.01	0.79	0.007	4.25 (0.14)	0.016 (0.004)	5.24 (0.09)	0.715 (0.015)
	NHD+S	1,267	0	0.24	0.55	0.005	4.28 (0.20)	0.013 (0.005)	6.01 (0.20)	0.771 (0.016)
	DDD+S	1,267	5.00	-0.01	0.62	0.007	4.34 (0.20)	0.015 (0.005)	6.51 (0.28)	0.754 (0.020)

NOTE. Each value was obtained by taking the mean among 100 networks generated by simulations. The number in parentheses represents the standard deviation calculated from the 100 networks.

a. See Table 1.

b. Parameters used in the simulations. See Methods.

Simulation studies showed that the value of *ν *increases (the slope becomes steeper) as *σ *decreases for both DDD+A and DDD+S (Figure 2C). We found that the disassortative structures of the yeast (MIPS), worm, and fly PINs were successfully reproduced by DDD+A and DDD+S when the values of *σ *are negative (Table 2, additional file 3: Figure S3). The human (Rual et al.) PIN was best regenerated by DDD+S with *σ *= 0. Note that, although *σ *= 0 means no degree-dependency of duplicability, where the DDD model becomes identical to the NHD model, the resultant network is still disassortative (Figure 2C). Therefore, in order to generate a network similar to the malaria parasite PIN, the value of *σ *has to be positive, *i.e.*, high-degree nodes should be duplicated more preferentially than low-degree nodes. In fact, our analysis showed that the assortativity of the malaria parasite PIN was reproduced by the DDD model with a positive *σ *(see Table 2 and additional file 3: Figure S3*E*).

The effect of link gains after gene duplication was also investigated. However, random attachments of links to duplicated nodes do not essentially affect the assortativity of resultant networks (additional file 4: Figure S4).

We also examined the average shortest path length, <*L*> and the extent of modularity, *M *in PINs (Table 1) and simulation-generated networks (Table 2). In agreement with our previous study [14], the values of <*L*> in the networks by NHD+A are larger than the actual values in PINs for all species. DDD+A gave the <*L*> values that are slightly closer to the actual values than NHD+A. On the other hand, for both NHD and DDD models, the symmetric divergence generated networks having larger values of <*L*>. It was reported that PINs are highly modular [32], but simulation-generated networks showed even higher values of *M *than the PINs (Table 2). Moreover, when we compare four networks generated by different models for each species, the value of *M *is positively correlated with that of <*L*>, which is consistent with Zhang and Zhang [33].

To see whether the difference in duplicability dependent on degrees accounts for the difference in assortativity, we analyzed orthologous relationships using proteomes in 55 eukaryote species. Wapinski et al. [34] provided data of orthologous relationships among 19 Ascomycota fungi including *S. cerevisiae*. In their dataset, all proteins in these 19 species are classified into ortholog groups, each of which consists of the proteins descended from a single ancestral protein in their most recent common ancestor. To evaluate the duplicability of a given gene in *S. cerevisiae*, we examined orthologous relationships between *S. cerevisiae *and each of the other 18 Ascomycota fungi. A phylogenetic tree was constructed using orthologous genes from the two species, and the number of gene duplication events observed in the phylogenetic tree was regarded as a duplicability of the gene (see Methods). In the same manner, we also evaluated gene duplicability in *C. elegans*, *D. melanogaster*, *H. sapiens*, and *P. falciparum *using other databases (see Methods).

**Figure 4:**
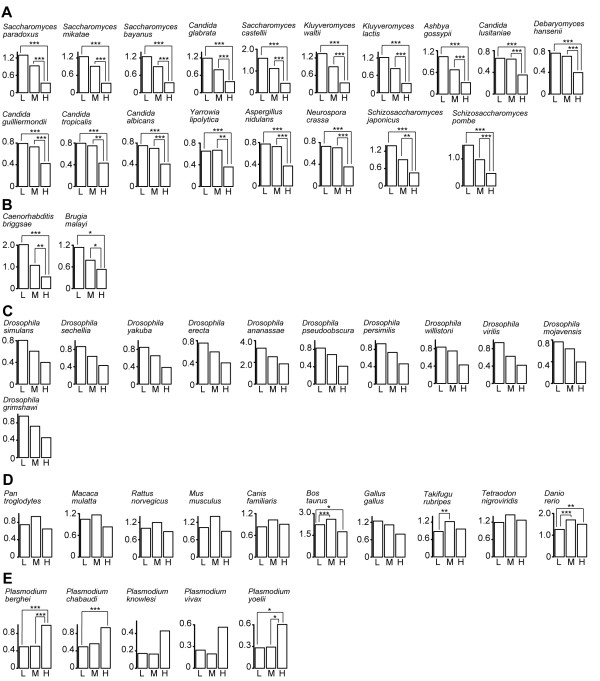
**Gene duplicability dependent on degrees**. Correlation between the degree and the duplicability of proteins in the **(*A*) **yeast, **(*B*) **worm, **(*C*) **fly, **(*D*) **human, and **(*E*) **malaria parasite PINs. L, M, and H represent low- (*k *= 1), middle- (*k *= 2-6), and high-degree (*k *> 7) proteins, respectively. A vertical axis indicates the mean duplicability in each category. A species name above each diagram denotes the species with which the orthologous relationships were examined. For example, in the top left diagram in **(*A*)**, gene duplicabilities were investigated using a phylogenetic tree containing *S. cerevisiae *and *S. paradoxus *genes. In **(*A*) **and **(*C*)**, the results for MIPS and Rual et al. datasets, respectively, are shown, and those for other yeast and human datasets are provided in Additional file 5: Figure S5. In each diagram, the duplicability of proteins in each category is compared to one another by using the Wilcoxon rank-sum test with the Bonferroni correction. *, *P *< 0.05; **, *P *< 0.01; ***, *P *< 0.001.

Figure 4 and additional file 5: Figure S5 indicate the relationships between the degree and the duplicability. We classified all proteins in each PIN into three categories containing similar numbers of proteins: low- (*k *= 1), middle- (*k *= 2 - 6), and high- (*k *> 6) degree proteins. The results showed that the duplicability of low- and middle-degree proteins is significantly higher than that of high-degree proteins in the yeast and worm PINs (Figure 4 and additional file 5: Figure S5). The same trend was also observed in the fly PIN. In contrast, the duplicability of low- and middle-degree proteins is significantly lower than that of high-degree proteins in the malaria parasite PIN, while no clear trends were observed in the human PIN (Figure 4). These observations are consistent with the above hypothesis; *i.e.*, the differences in degree-dependent duplicability of genes account for the difference in assortativity among species.

We also investigated the differences in degrees and duplicabilities among different functional categories in yeast and malaria parasite proteins. Table 3 shows the mean degree and the mean duplicability of yeast proteins belonging to each category obtained from the GO (gene ontology) slim database in the Saccharomyces Genome Database [3]. Interestingly, genes in several categories with significantly higher (lower) degrees on average showed significantly lower (higher) duplicabilities. A similar analysis was conducted for malaria parasite proteins using the GO in the PlasmoDraft database [35] (Table 4). In this case, functional categories with high (low) degrees tend to show high (low) duplicabilities (additional file 6: Figure S6), which is an opposite trend to that observed in yeast proteins. The slopes in the degree-duplicability relationships are significantly different between the yeast and malaria parasite PINs (*P *< 0.01; analysis of covariance).

**Table 3:** Degrees and duplicabilities of the genes in the yeast PIN belonging to each functional category

GO biological process category	**Mean degree**^**a**^	**Mean duplicability**^**a**^	# of proteins
Lipid metabolic process	2.2	0.79 (---)	127
Cellular respiration	2.2	0.34	57
Vitamin metabolic process	2.6	1.33 (+)	51
Cofactor metabolic process	2.6	0.72	86
Translation	2.6	0.70	167
Aromatic compound metabolic process	2.9	0.50	42
Heterocycle metabolic process	2.9	0.40	48
Carbohydrate metabolic process	3.1	1.80 (+++)	97
Amino acid and derivative metabolic process	3.6	0.81	124
Generation of precursor metabolites and energy	3.9	0.86	95
Sporulation	4.0	0.66	84
Cell wall organization and biogenesis	4.1	1.42 (+++)	129
Response to chemical stimulus	4.1 (+)	1.05 (+++)	271
Protein modification process	4.1 (++)	0.72	368
Cellular homeostasis	4.2	1.12	63
DNA metabolic process	4.5	0.42 (---)	244
Protein catabolic process	4.7 (+)	0.31 (---)	119
Meiosis	4.7 (++)	0.27 (-)	104
Transport	4.8 (+++)	1.93 (+)	649
Response to stress	4.8 (+++)	0.72	348
Organelle organization and biogenesis	4.9 (+++)	0.46 (---)	947
Vesicle-mediated transport	5.3 (+++)	0.56	261
Cell cycle	5.3 (+++)	0.49	292
Pseudohyphal growth	5.4	0.83	50
Signal transduction	5.6 (+++)	1.08 (+)	168
Membrane organization and biogenesis	5.7 (+++)	0.64	149
RNA metabolic process	6.0 (+++)	0.18 (---)	496
Cytokinesis	6.1 (+++)	0.62	91
Transcription	6.3 (+++)	0.22 (---)	178
Conjugation	6.4 (+++)	0.71	89
Anatomical structure morphogenesis	6.8 (+++)	0.63	115
Cell budding	7.5 (+++)	0.78	70

All proteins in the yeast PIN	3.7	0.92	3,891

NOTE. Functional categories containing five or more proteins are shown. Genes in the MIPS database were used.

a. The mean among the proteins contained in each functional category. +++, ++, and + (or ---, --, and -) indicates that a given value is significantly higher (or lower) with *P *< 0.001, *P *< 0.01, and *P *< 0.05, respectively, by the Wilcoxon rank-sum two-sample test with the Bonferroni correction.

**Table 4:** Degrees and duplicabilities of the genes in the malaria parasite PIN belonging to each functional category

GO biological process category	**Mean degree**^**a**^	**Mean duplicability**^**a**^	**# of proteins**^**a**^
Pathogenesis	5.6	2.21	42
Interaction with host	5.0	2.15	42
Multi-organism process	5.0	1.18	93
Proteolysis	4.9	0.99	20
Cellular catabolic process	4.8	0.09	19
Symbiosis encompassing mutualism through parasitism	4.6	1.27	86
Ubiquitin-dependent protein catabolic process	4.4	0.07	25
Cellular component assembly	4.3	0.59	22
Biological adhesion	4.3	1.10	26
Localization	4.2	0.10	13
Translation	4.1	0.16 (-)	115
Gene expression	4.0	0.26	294
Cellular macromolecule catabolic process	4.0	0.08	26
RNA metabolic process	3.9	0.31	159
Macromolecule catabolic process	3.8	0.07	29
Protein catabolic process	3.8	0.07	26
Biosynthetic process	3.7	0.21	127
Biopolymer catabolic process	3.7	0.06	25
Response to stimulus	3.5	0.15	28
Nucleobase, nucleoside, nucleotide and nucleic acids metabolism	3.4 (--^a^)	0.28	147
Ribonucleoprotein complex biogenesis and assembly	3.4	0.19	20
Post-translational protein modification	3.1	1.33	8
Antigenic variation	3.1	0.38	38
Cellular component organization	3.1	0.24	26
Transport	2.1	0.16	8

All proteins in the malaria parasite PIN	4.3	0.42	1,267

NOTE. Functional categories containing five or more proteins are shown.

a. See Table 3.

## Discussion

### Disassortative structures in PINs

In this paper, we showed that the yeast, worm, fly, and human PINs are disassortative, while the malaria parasite PIN is not disassortative. Therefore, a disassortative structure is not a common feature of PINs. By comparing proteomes and conducting simulations, we demonstrated that the difference in assortativity can well be explained by assuming that the duplicability of proteins is dependent on its degree and the dependency is different among species. If low-degree proteins have preferentially duplicated in evolution as in yeast, worm, and fly, or there is no trend in the duplicability between low- and high-degree proteins as in the human, the PIN becomes disassortative. On the other hand, a PIN without a disassortative structure could be generated if high-degree proteins have preferentially duplicated as in malaria parasite. Therefore, for explaining the presence of a disassortative structure in PINs, the "selectionist view" as proposed by Maslov and Sneppen [17] is not necessary. It is rather likely that a disassortative structure observed in PINs is merely a byproduct of preferential duplications of low-degree proteins.

Although several authors [25,27] claimed that the suppression of hub-hub interactions may be an artifact, our analyses using four recently published high-quality yeast PIN datasets demonstrated that all of the four PINs are in fact disassortative. In Batada et al. [27], they mentioned that the interactions between hubs are not suppressed, where a hub was defined as a node with *k *> 21 (top 10% of the nodes). However, the same data showed that the interactions between nodes with relatively high degrees (20 <*k *< 30) and those with very high degrees (*k *> 50) are suppressed and interactions between low-degree nodes (*k *< 3) and high-degree nodes (*k *> 50) are favored. Therefore, Batada et al.'s data [27] is not inconsistent with the presence of a disassortative structure. Moreover, the updated version [30] of their multi-validated yeast PIN data clearly showed disassortativity (see additional file 2: Figure S2*A*). These results suggest that a disassortative structure in the yeast PIN is not an artifact.

Fernández [36] classified yeast proteins into several categories on the basis of the existence of orthologous proteins in other genomes, e.g., the proteins that are present in eukaryotes, eubacteria, and archaebacteria, or those present in other fungi. He found that an "ancient" network consisting of proteins that are present in diverse organisms tends to be assortative and the assortative ancient network evolved into the disassortative PIN in yeast at the present time. To explain this evolutionary trend, Fernández [36] hypothesized a model in which an attachment of new links between similar-degree nodes is disfavored. Note that our DDD model is also consistent with the evolutionary trend toward higher disassortativity (see additional file 7: Figure S7).

PIN data include binary interaction information that is directly obtained from experiments such as Y2H and indirectly inferred from protein complex data. Wang and Zhang pointed out that these two types of data may give quite different images of PINs [32]. We therefore excluded protein complex data from the MIPS database and reexamined the yeast PIN. The result, however, showed no significant differences in disassortativity between PINs with and without complex data (additional file 8: Figure S8). We should also note that PINs are a collection of potential interactions that occur at different times in different cells or subcellular locations, but we treated all interactions simultaneously. To see how such treatment affects our results, we examined yeast subnetworks constructed from the proteins in each subcellular localization separately. As shown in additional file 9: Figure S9, although the extent of disassortativity varies among different subcellular locations due to smaller sample sizes, in general such subnetworks also show disassortative structures.

### Neofunctionalization and subfunctionalization

It is generally thought that gene duplication is a primary source for generating organismal complexity. Neofunctionalization and subfunctionalization are proposed as a fate of duplicated genes. Neofunctionalization hypothesizes that the presence of redundant copies of genes allows one duplicate to be free from selective pressure, and thus one of the duplicates can accumulate random mutations and potentially acquire novel functions [37]. Subfunctionalization argues that each of the duplicates accumulates degenerative mutations, resulting in the division of ancestral functions into complementary subsets [38]. Both neofunctionalization and subfunctionalization contribute to protein evolution [39-42].

In the duplication-divergence model, neofunctionalization and subfunctionalization are modeled as a random attachment of new links [20] and a random loss of links to duplicated nodes [22], respectively. Our simulation studies showed a high rate of link losses (α > 0.5; see Table 2), suggesting the importance of subfunctionalization. On the other hand, link gains were shown to have only minor effects to the structure of PINs (additional file 4: Figure S4). Altogether, our study supports a view that subfunctionalization plays a significant role in shaping the structures of PINs, which is consistent with a recent study by Gibson and Goldberg [43].

As for subfunctionalization, it has been reported that the number of links retained after gene duplication is considerably different between two duplicates [44]. For this reason, several previous studies used the asymmetric divergence model [14,45-48]. However, "complete" asymmetric divergence in which links are eliminated from only one of the duplicates is unrealistic, and the actual situation should be between asymmetric divergence and symmetric divergence. We have therefore conducted simulation studies using both symmetric and asymmetric divergence models. The results, however, did not show essential differences (Table 2).

### Degree-duplicability correlations

In this study, we found that lower-degree proteins tend to duplicate more frequently in the yeast, worm, and fly PINs (Figure 4). One caveat of this analysis is that the degrees of proteins used in these analyses are present-day degrees and thus might be different from those prior to duplication. Because the number of interactions often changes greatly after duplications [19,41], the observed degree-duplicability correlation may also be interpreted as that degrees decrease after duplication by divergence rather than that the duplicability itself is dependent on a degree. However, under this interpretation, it is difficult to explain the difference in the trend of degree-duplicability correlations among different species (Figure 4). Moreover, as mentioned above, the duplication-divergence model without considering degree-dependent duplicability is insufficient to explain the extent of disassortativity in the yeast, worm, and fly PINs.

Prachumwat and Li [49] found a positive correlation between degree and the proportion of unduplicated proteins in the yeast proteome, which is consistent with our results. Liang et al. [50] showed that the extent of protein under-wrapping, which indicates the solvent accessibility to backbone hydrogen bonds, is negatively correlated with gene duplicability in *Escherichia coli*, yeast, worm, fly, human, and *Arabidopsis thaliana*. They also found that the correlation becomes weaker for more complex organisms. It was reported that the extent of protein under-wrapping is strongly positively correlated with the degree of proteins in yeast [51]; therefore, their results are also consistent with ours (Figure 4). In Liang et al. [50], gene duplicability was defined as a protein family size. In this study, we evaluated gene duplicability by directly counting the number of gene duplication events using orthologous genes in closely related species. Therefore, we considered more recent gene duplications than Prachumwat and Li [49] and Liang et al. [50]. He and Zhang showed that low-degree nodes are less important [52] and less important genes tend to duplicate more frequently [53]. Their results are also consistent with ours.

Why low-degree proteins tend to be duplicated frequently in the evolution of the yeast PIN? The actual reason is currently unclear. Yet, as indicated in Table 3, some functional categories showed low degrees but high duplicabilities on average, while others showed high degrees and low duplicabilities. The former includes metabolic processes for carbohydrates or vitamins. Marland et al. [54] reported that the duplicability of genes involved in metabolism, especially in central metabolism, is significantly higher than that for non-metabolic genes in both yeast and *E. coli*. Moreover, most of the enzymes involved in these metabolic processes bind only to a specific substrate, and probably for this reason, their degrees are relatively low. The categories showing a high degree and a low duplicability are exemplified by organelle organization and biogenesis, RNA metabolic process, and transcription (see Table 3). The category "organelle organization and biogenesis" contains many proteins involved in the organization of actin filaments or cytoskeletons. Actin and actin-related proteins are known to bind many partner proteins [55]. At the same time, they are highly conserved from yeasts to humans [56], and therefore gene duplications of these genes are apparently rare.

Why, then, are high-degree proteins duplicated preferentially in the evolution of the malaria parasite PIN? Table 4 indicates that genes belonging to the categories pathogenesis and interaction with host tend to have high degrees and high duplicability, though the numbers of genes in these categories are not large. These categories include many proteins of Pf erythrocyte membrane protein 1 (PfEMP1) family. PfEMP1 proteins interact with receptors in the host and change the morphology of the host cell [57]; therefore, the duplications of these genes would be beneficial to malaria parasites. Moreover, a PfEMP1 protein has a feature of an adhesive molecule [58] and can bind many partner proteins. However, the actual reason for the opposite trend of gene duplicability in the entire PIN of malaria parasite to that of other eukaryotes is currently unclear. It would be intriguing to investigate the PINs of other parasitic organisms.

These observations suggest that the duplicability of the proteins having a given function can be different and determined by each organism's living environment. The duplicability of genes for each species would, in turn, determine the overall structure of a PIN. The availability of high-quality interaction data from various species including parasitic organisms will help us to clarify the relationships between environments where organisms inhabit and the evolution of their PINs in greater detail.

## Conclusions

In this study, we showed that disassortative structures are not common features among eukaryotes by examining nine different PINs from five eukaryote species. We found that low-degree proteins tend to show high duplicabilities for the PIN with a disassortative structure (*e.g. *yeast), while an opposite trend was observed for the PIN without disassortativity (*e.g. *malaria parasite). Simulation studies on the basis of gene duplication and divergence also supported these observations. Therefore, for explaining the presence of disassortative structure, any selective forces on the entire structure of PINs are unnecessary. Our results indicate that overall structure of PINs is primarily determined by local processes in the course of evolution.

## Methods

### PIN and GO data

The datasets of the yeast PIN were obtained from the MIPS (Munich Information Center for Protein Sequences) database http://mips.gsf.de (18 May 2006) [3], Batada et al. [30], Reguly et al. [29], and Yu et al. [4]. Worm and Fly PIN data were obtained from Li et al. [5] and IM Browser http://proteome.wayne.edu/PIMdb.html[59], respectively. The datasets of the human PIN were from Rual et al. [7] and Stelzl et al. [8], and Malaria parasite PIN was from LaCount et al. [9]. Some of these datasets contain components that are not connected to each other. In these cases, we used the largest component for the analysis. All self-interactions were removed. The yeast GO slim dataset was downloaded from the ftp site of Saccharomyces Genome Database ftp://genome-ftp.stanford.edu/pub/yeast/literature_curation/. The GO dataset for *P. falciparum *was obtained from PlasmoDraft [35]. The yeast PIN excluding protein complex data was obtained from http://www.umich.edu/~zhanglab/download.htm[32].

### Modularity

PINs have a modular structure, in which interactions between proteins are much denser within a module than between modules [32]. The modularity *m *for a particular separation of a network is calculated by $m=\sum_{s=1}^{N}\left[(l_{s}/L)-{\left(k_{s}/2L\right)}^{2}\right]$, where *N *is the number of modules, *L *is the number of links in a network, *l*_s _is the number of links within module *s*, and *k_s _*is the sum of the degrees of nodes in module *s *[60]. The separation that maximizes *m *is considered to be optimal. The maximum *m *among all possible separation of a given network is referred to as the modularity of the network and denoted as *M*. We used the method by Vincent et al. [61] for searching the optimal separation, since the method gives excellent accuracy for module separation and outperforms other methods in terms of a computational time [61].

### Simulation

The simulation studies were conducted using a duplication-divergence model in a similar manner to Hase et al. [14] with a modification. In the DDD model, a new node and new links are added to the network according to the following rules at each time step of a simulation. (1) A node in a network is randomly selected (A). Node A is duplicated to generate a new node (A') with a probability (1 + *σk*)/1,000 (when 1 + *σk *>0), where *k *is the degree of node A, and *σ *is a parameter determining the duplicability of a node for each species. The probability is defined to be 0 when 1 + *σk *is lower than 0. The interacting pattern of node A' is identical to that of node A. (2) For a divergence process, two different models were examined: the asymmetric divergence [14] and the symmetric divergence (Figure 3). In the former, links to node A' is removed with a uniform probability *α*. In the latter, for each of the nodes connecting to A and A' (e.g. node B), one of the two links (either A-B link or A'-B link) is randomly chosen and is removed with a probability *α *(Figure 3). (3) A new link between node A and node A' is created with a probability *βn*_N _(when *βn*_N _≤ 1), where *n*_N _is the number of common neighbors shared by these two nodes. The probability is defined to be one when *βn*_N _is greater than 1. If there are no links to node A' after these processes (all links to node A' were removed and no links were generated), node A' is not added to the network.

The processes (1)-(3) were repeated until the number of nodes in a network became the same as those in the PINs for a given species. We used various values of *σ*, *α*, and *β *and performed simulations. The value of *σ *was changed from -0.05 to 0 by 0.01 and from 0 to 10.0 by 0.1, and the values of *α *and *β *were changed from 0 to 1 by 0.01 and 0.001, respectively. For a given set of *σ*, *α*, and *β*, we conducted simulations 100 times. We then calculated the mean of <*k*> and the mean of <*C*> from the 100 networks. Moreover, we calculated the mean of <*K*_nn_(*k*)> from the 100 networks. The value of *ν *represents the slope of the regression line of the mean of <*K*_nn_(*k*)>. In Table 2, the values of *σ*, *α*, and *β *that could reproduce <*k*>, <*C*>, and *ν *in each PIN are shown.

We also examined a model considering link gains. In this model, the following process was added after the process (3) in the DDD model: A link is attached between each of the two duplicated nodes (A and A') and a randomly selected node with a probability *ε*. The value of *ε *was changed from 0.01 to 0.1. *σ *= -0.05 was used for both asymmetric and symmetric divergence. The values of *α *and *β *were determined in the same way as the DDD model.

### Gene duplicability

We examined the duplicability of genes in yeast, worm, fly, human, and malaria parasite by using orthologous relationships among closely related species. For yeast genes, we used the dataset of ortholog groups for 19 Ascomycota fungi including *S. cerevisiae *downloaded from Fungal Orthogroups Repository http://www.broad.mit.edu/regev/orthogroups/[34]. This dataset provides ortholog groups, each of which consists of genes descended from a gene in the last common ancestor of 19 Ascomycota fungi. Duplicability of genes in the yeast PIN was evaluated by considering orthologous relationships between *S. cerevisiae *and each of the other 18 fungal species. Let us consider the comparison between *S. cerevisiae *and *S. paradoxus*, for instance. Because some ortholog groups do not contain any genes from some of the 19 species, we consider only ortholog groups containing at least one gene from both *S. cerevisiae *and *S. paradoxus*. Suppose that a given ortholog group contains two genes from *S. cerevisiae *and three genes from *S. paradoxus *(and more from other species). We constructed a phylogenetic tree from these five genes by the neighbor-joining (NJ) method [62] using ClustalW [63]. We then counted the number of duplication events from the tree using Notung (ver. 2.5) [64]. This number is regarded to be duplicabilities for both of two *S. cerevisiae *genes. In this way, the value of duplicability was assigned to each protein in the yeast PIN. Similarly, we calculated duplicability of genes contained in the worm, fly, human, and malaria parasite PINs. For worm and malaria parasite genes, we used OrthoMCL-DB version 2 http://orthomcl.cbil.upenn.edu[65], which contains ortholog groups of three nematode species including *C. elegans *and those of six Haemosporidian species including *P. falciparum*. For fly and human genes, we used ortholog groups of 12 *Drosophila *species and those of 11 vertebrate species including seven mammals, respectively, downloaded from OrthoDB http://cegg.unige.ch/orthodb[66].

## List of abbreviations

DDD: degree-dependent duplication; NHD: Non-uniform heterodimerization; PIN: protein-protein interaction network

## Authors' contributions

TH, YN, and HT designed the study; TH analyzed data and performed simulation studies; TH and YN wrote the paper. All authors read and approved the final manuscript.

## Supplementary Material

Additional file 1 **Figure S1**: **Degree distribution in the yeast and human PINs**. **(*A*) **Degree distribution *P*(*k*) in the yeast PIN for four different datasets. A dashed line is the same as Figure 1. **(*B*) **Degree distribution *P*(*k*) in the human PIN for two datasets. A dashed line is the same as Figure 1. Click here for file

Additional file 2 **Figure S2**: **<*K*_nn_(*k*)> in the yeast and human PINs**. **(*A*) **<*K*_nn_(*k*)> in the yeast PIN for four different datasets. Dashed lines in black, blue, green, and red represent *k*^-0.47^, *k*^-0.33^, *k*^-0.33^, and *k*^-0.25^, respectively. **(*B*) **<*K*_nn_(*k*)> in the human PIN for two datasets. Dashed lines in black and red represent *k*^-0.26 ^and *k*^-0.27^, respectively. Click here for file

Additional file 3 **Figure S3**: **Distribution of <*K*_nn_(*k*)> in the PINs and the networks generated by the NHD and DDD models**. Distribution of <*K*_nn_(*k*)> in the PIN (black square) and the networks by DDD+A (red diamond), DDD+S (blue triangle), NHD+A (green cross), and NHD+S (purple plus) for **(*A*) **yeast, **(*B*) **worm, **(*C*) **fly, **(*D*) **human, and **(*E*) **malaria parasite. The results for the NHD and DDD models were obtained by taking the mean among 100 networks generated by simulations. A dashed line represents a regression line. The slope (*ν*) of each regression line is shown in Table 2. Click here for file

Additional file 4 **Figure S4**: **Distribution of <*K*_nn_(*k*)> in the networks generated by simulations with link gains for (*A*) the DDD+A and (*B*) DDD+S models**. *ε *is the probability of a link gain (see Methods). The results were obtained by taking the mean among 100 networks generated by simulations. A dashed line represents a regression line (*ν *= 0.51 and 0.48 for the asymmetric and symmetric divergence, respectively). Click here for file

Additional file 5 **Figure S5**: **Gene duplicability dependent on degree in the yeast and human PINs**. Duplicability of genes in the yeast and human PINs for **(*A*) **Batada et al., **(*B*) **Reguly et al., **(*C*) **Yu et al., and **(*D*) **Stelzl et al. Click here for file

Additional file 6 **Figure S6**: **Relationships between mean degrees and mean duplicabilities for different functional categories in (*A*) yeast and (*B*) malaria parasite**. A dot indicates each functional category, and its size represents the number of proteins in the category. A dashed line indicates a regression line. Click here for file

Additional file 7 **Figure S7**: **Evolutionary trend toward higher disassortativity in the networks generated by the DDD model**. Fernández [36] categorized yeast proteins into five classes: proteins that are present in all organisms (3.5% of the yeast proteome), in eubacteria (9.5%), in archaebacteria but not in eubacteria (8%), in eukaryotes diverging earlier than fungi (19%), in other fungi (36%), and exclusively in yeast (24%). By using these fractions, we calculated the numbers of nodes contained in ancient networks as 136, 505, 1,556, and 3,268. We generated networks by the DDD model (asymmetric divergence) with *σ *= -0.05, *α *= 0.50, and *β *= 0.019, which were used for regenerating the yeast PIN (see Table 1). For each ancient network, we calculated the mean value of *ν *from 100 simulation-generated networks. Click here for file

Additional file 8 **Figure S8**: **Disassortative structure in the yeast PIN with and without protein complex data**. Distribution of <*K*_nn_(*k*)> in the yeast PIN with (black square) and without protein complex data (red triangle). Click here for file

Additional file 9 **Figure S9**: **Disassortative structures of the yeast sub-PINs constructed from proteins in different subcellular localizations**. *ν *= 0.40, 0.48, 0.29, 0.17, and 0.10 for cytoplasm, cell periphery, punctate composite, nucleolus, and nucleus, respectively. The subcellular localization data were downloaded from http://www.umich.edu/~zhanglab/download/Wang_PLoSCB_Suppl/description.htm. Subcellular localizations containing >100 proteins and >30 interactions were shown. Click here for file
