# Supplementary figures and images for: Difference in gene duplicability may explain the difference in overall structure of protein-protein interaction networks among eukaryotes
Source: BMC Evol Biol. 2010 Nov 18;10:358. doi: 10.1186/1471-2148-10-358 (PMC2994879; doi:10.1186/1471-2148-10-358)

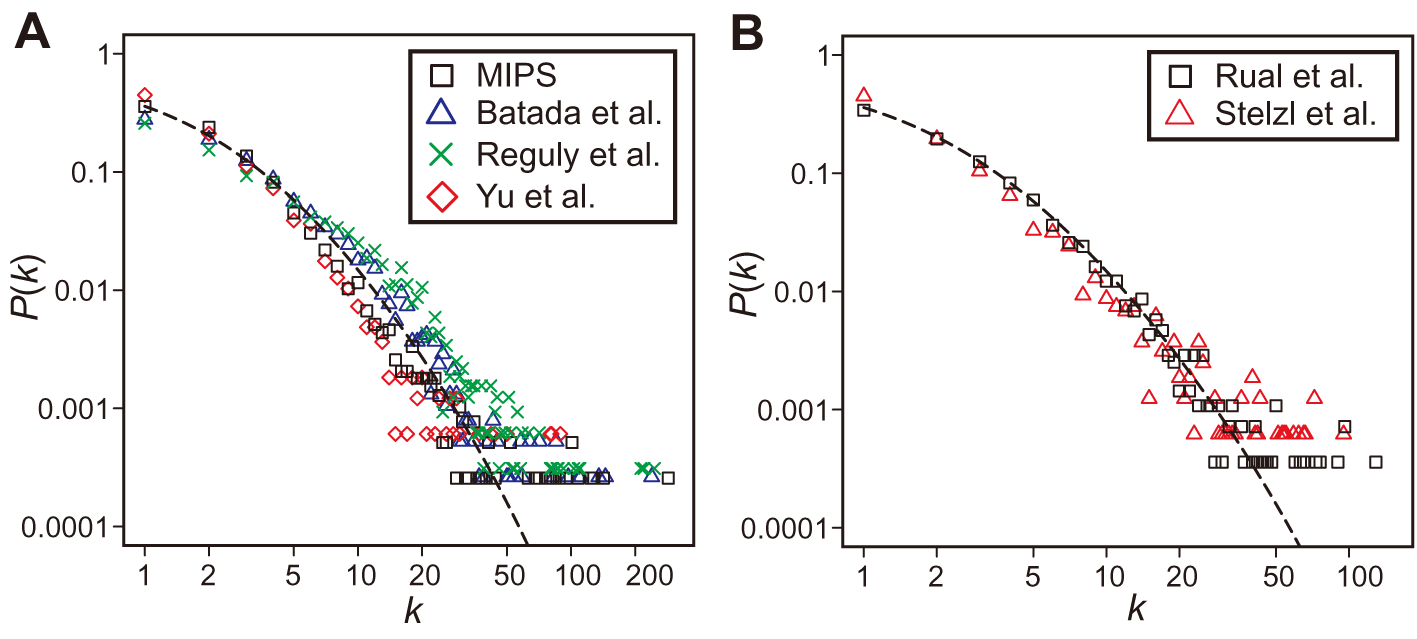

Supplement: Additional file 1 — Figure S1: Degree distribution in the yeast and human PINs. (A) Degree distribution P(k) in the yeast PIN for four different datasets. A dashed line is the same as Figure 1. (B) Degree distribution P(k) in the human PIN for two datasets. A dashed line is the same as Figure 1. [file 1471-2148-10-358-S1.TIFF]

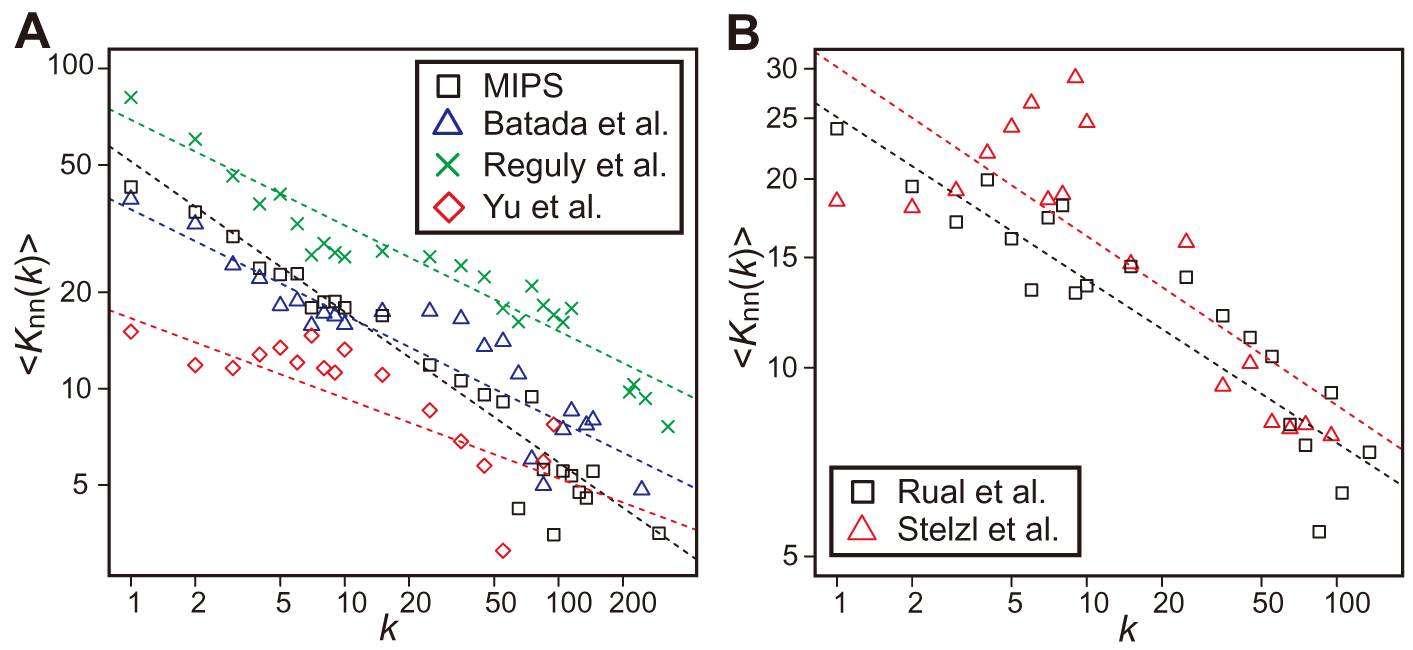

Supplement: Additional file 2 — Figure S2: <Knn(k)> in the yeast and human PINs. (A) <Knn(k)> in the yeast PIN for four different datasets. Dashed lines in black, blue, green, and red represent k-0.47, k-0.33, k-0.33, and k-0.25, respectively. (B) <Knn(k)> in the human PIN for two datasets. Dashed lines in black and red represent k-0.26 and k-0.27, respectively. [file 1471-2148-10-358-S2.TIFF]

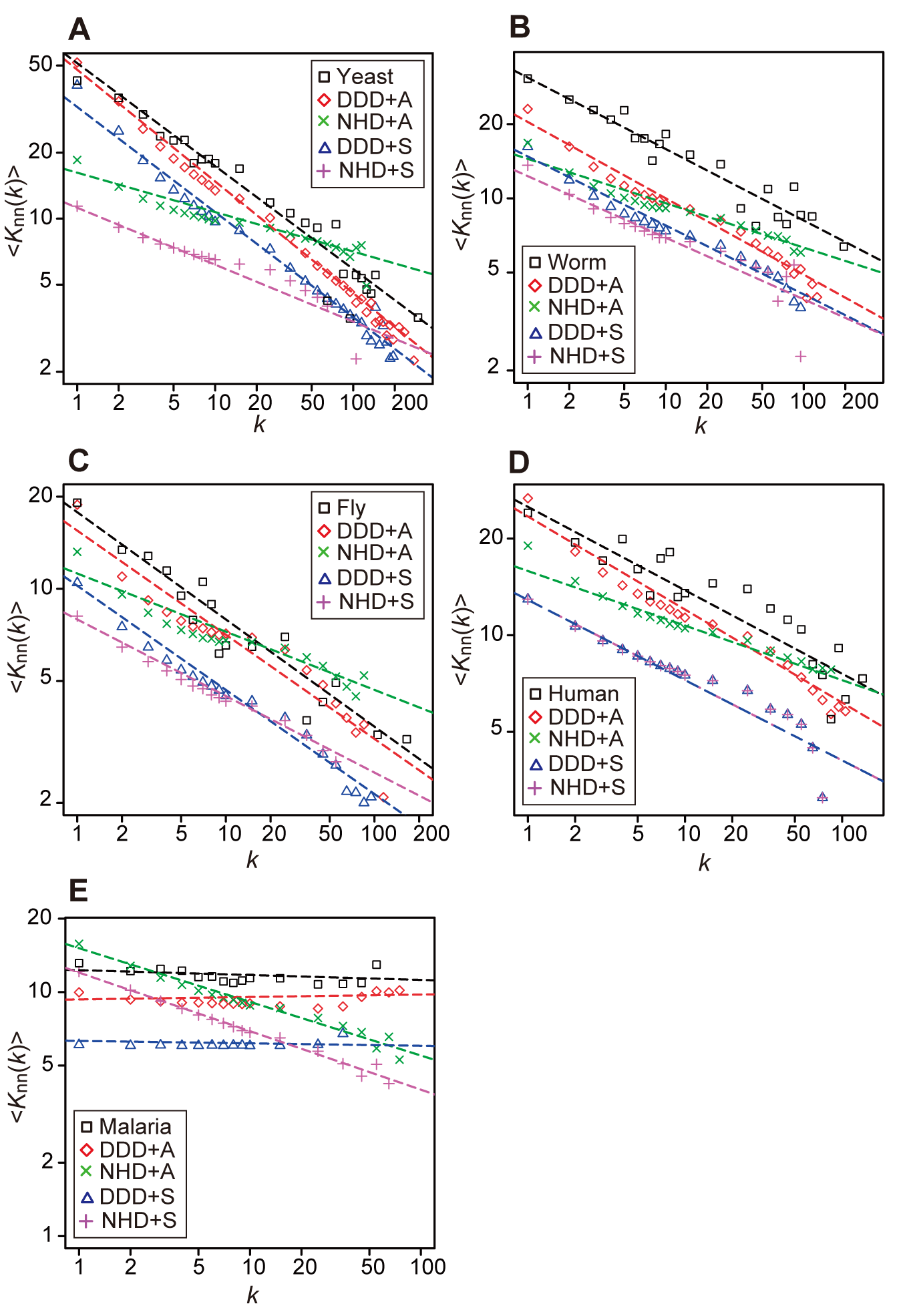

Supplement: Additional file 3 — Figure S3: Distribution of <Knn(k)> in the PINs and the networks generated by the NHD and DDD models. Distribution of <Knn(k)> in the PIN (black square) and the networks by DDD+A (red diamond), DDD+S (blue triangle), NHD+A (green cross), and NHD+S (purple plus) for (A) yeast, (B) worm, (C) fly, (D) human, and (E) malaria parasite. The results for the NHD and DDD models were obtained by taking the mean among 100 networks generated by simulations. A dashed line represents a regression line. The slope (ν) of each regression line is shown in Table 2. [file 1471-2148-10-358-S3.TIFF]

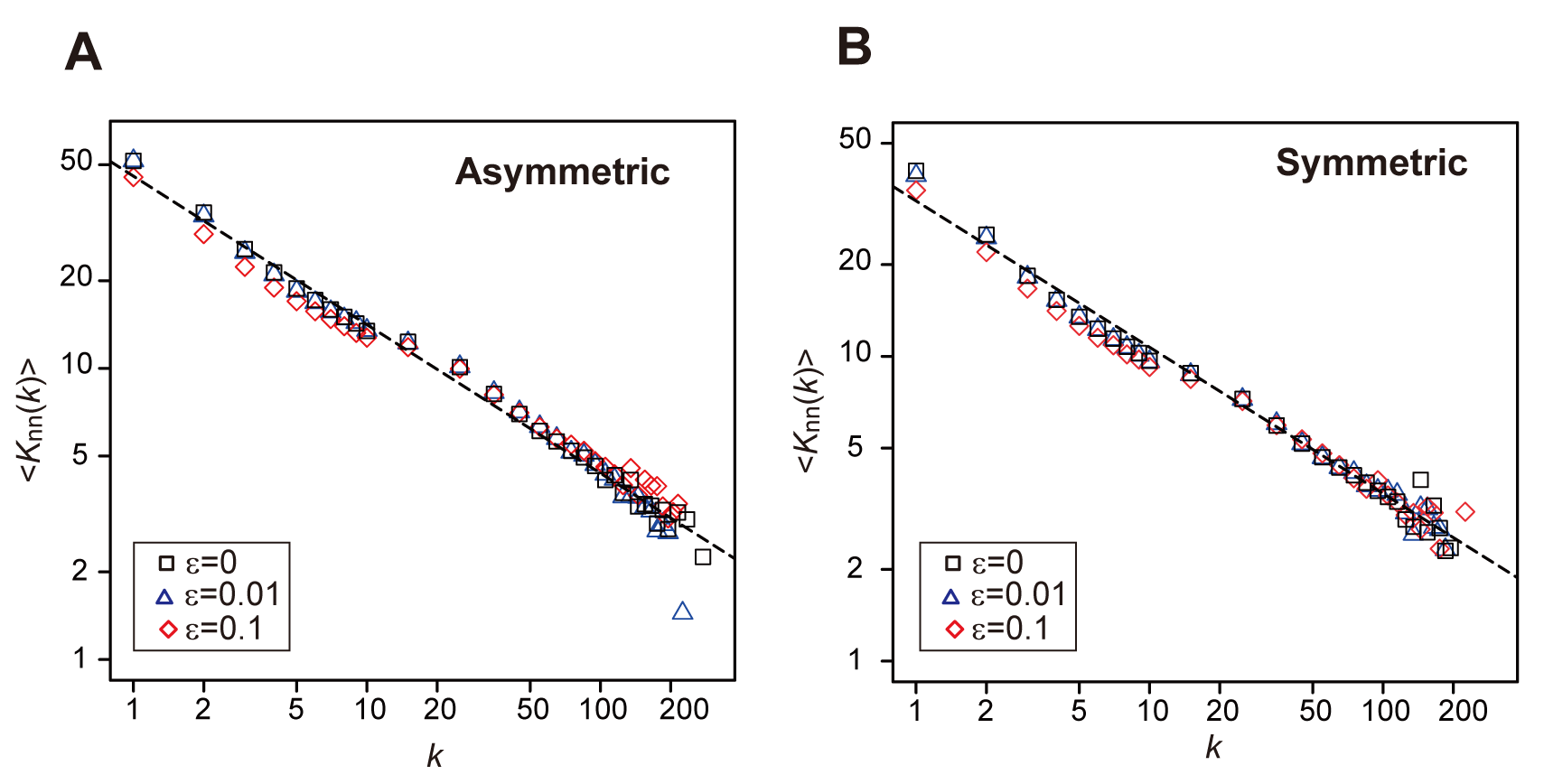

Supplement: Additional file 4 — Figure S4: Distribution of <Knn(k)> in the networks generated by simulations with link gains for (A) the DDD+A and (B) DDD+S models. ε is the probability of a link gain (see Methods). The results were obtained by taking the mean among 100 networks generated by simulations. A dashed line represents a regression line (ν = 0.51 and 0.48 for the asymmetric and symmetric divergence, respectively). [file 1471-2148-10-358-S4.TIFF]

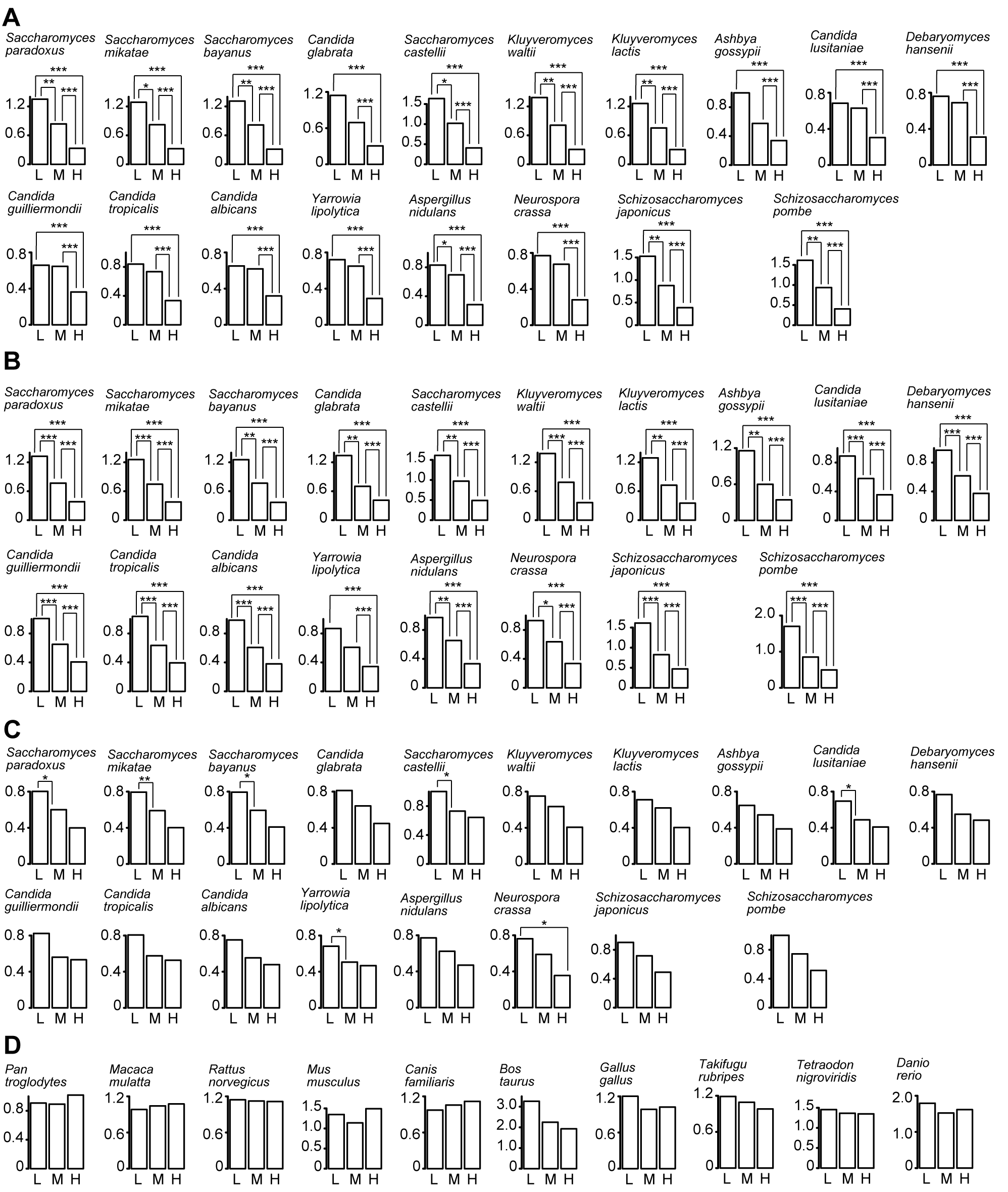

Supplement: Additional file 5 — Figure S5: Gene duplicability dependent on degree in the yeast and human PINs. Duplicability of genes in the yeast and human PINs for (A) Batada et al., (B) Reguly et al., (C) Yu et al., and (D) Stelzl et al. [file 1471-2148-10-358-S5.TIFF]

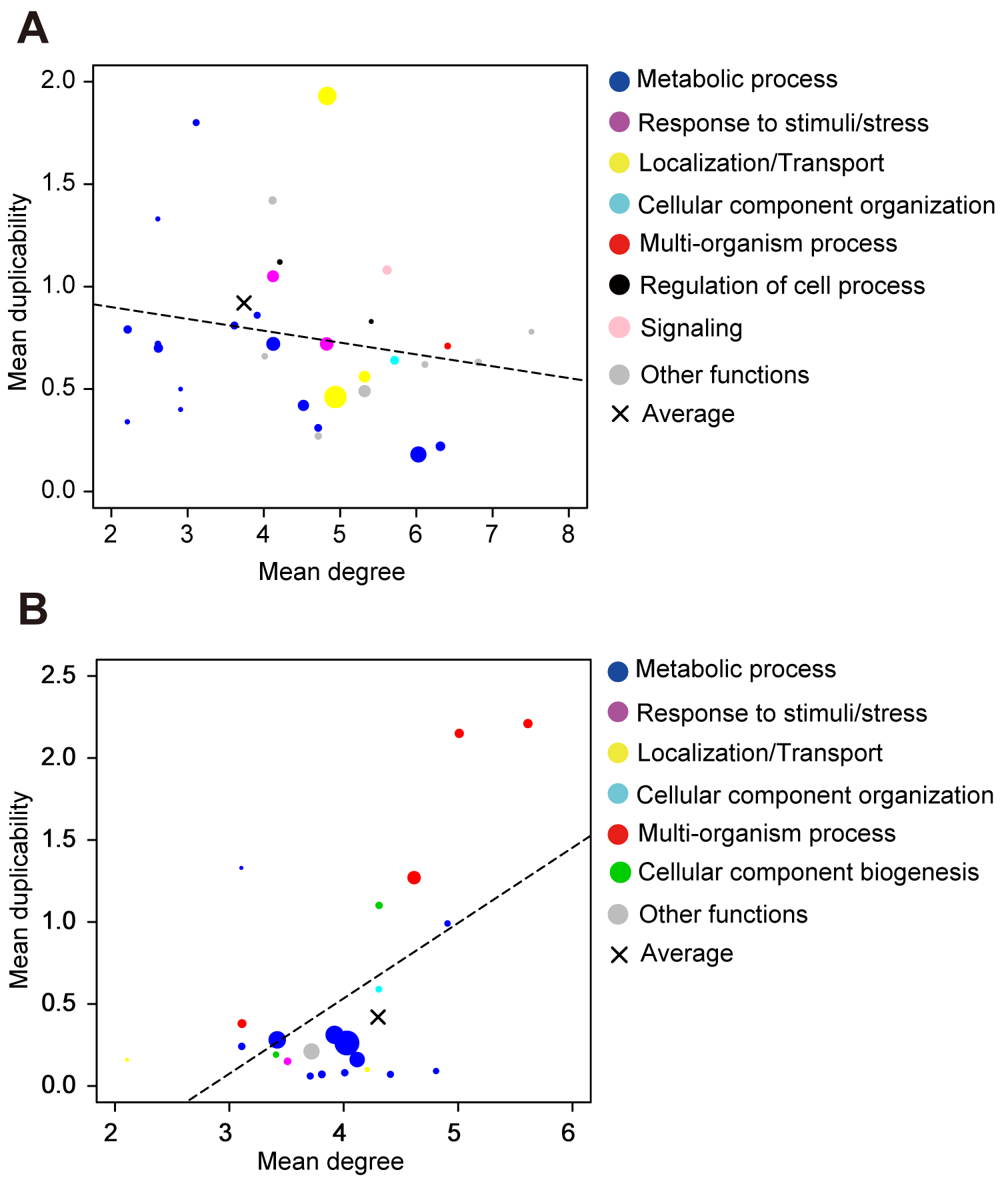

Supplement: Additional file 6 — Figure S6: Relationships between mean degrees and mean duplicabilities for different functional categories in (A) yeast and (B) malaria parasite. A dot indicates each functional category, and its size represents the number of proteins in the category. A dashed line indicates a regression line. [file 1471-2148-10-358-S6.TIFF]

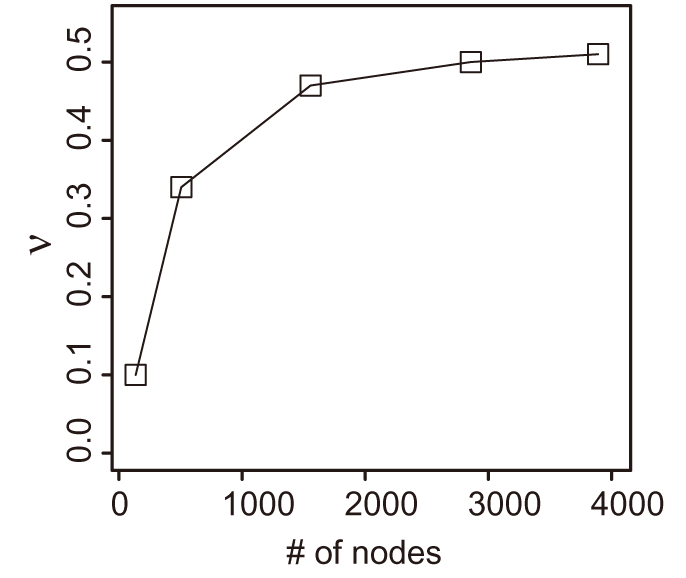

Supplement: Additional file 7 — Figure S7: Evolutionary trend toward higher disassortativity in the networks generated by the DDD model. Fernández [36] categorized yeast proteins into five classes: proteins that are present in all organisms (3.5% of the yeast proteome), in eubacteria (9.5%), in archaebacteria but not in eubacteria (8%), in eukaryotes diverging earlier than fungi (19%), in other fungi (36%), and exclusively in yeast (24%). By using these fractions, we calculated the numbers of nodes contained in ancient networks as 136, 505, 1,556, and 3,268. We generated networks by the DDD model (asymmetric divergence) with σ = -0.05, α = 0.50, and β = 0.019, which were used for regenerating the yeast PIN (see Table 1). For each ancient network, we calculated the mean value of ν from 100 simulation-generated networks. [file 1471-2148-10-358-S7.TIFF]

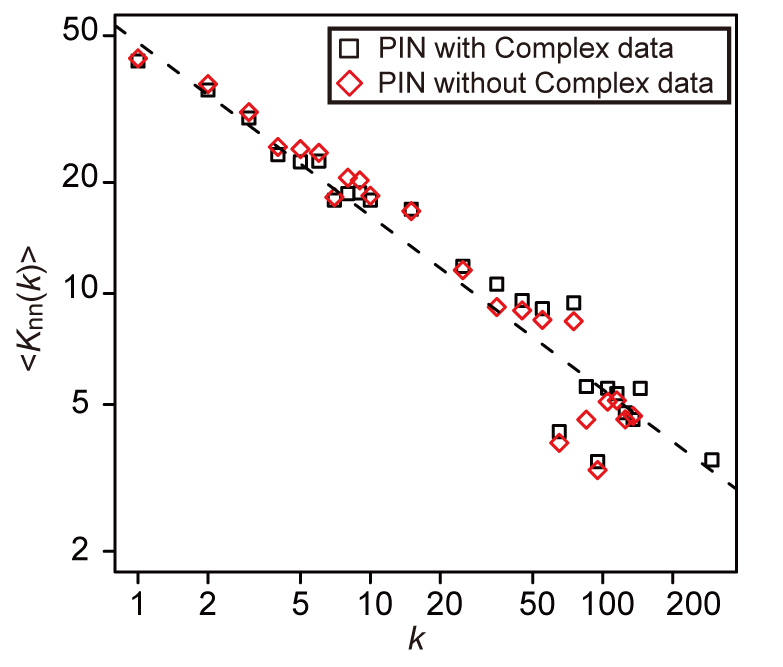

Supplement: Additional file 8 — Figure S8: Disassortative structure in the yeast PIN with and without protein complex data. Distribution of <Knn(k)> in the yeast PIN with (black square) and without protein complex data (red triangle). [file 1471-2148-10-358-S8.TIFF]

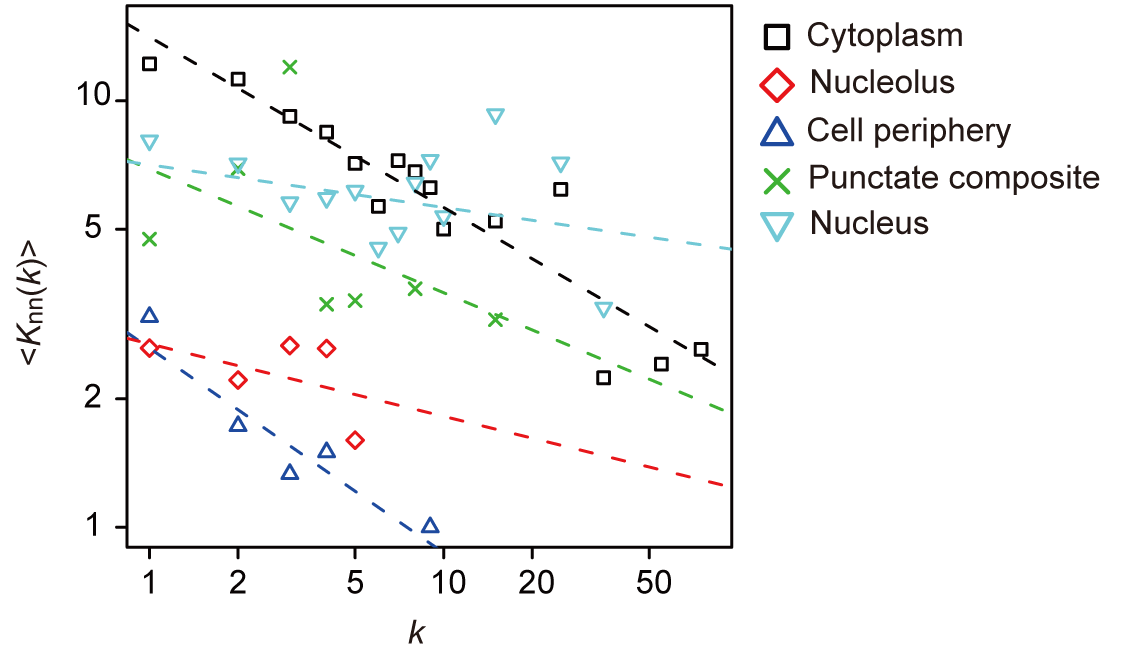

Supplement: Additional file 9 — Figure S9: Disassortative structures of the yeast sub-PINs constructed from proteins in different subcellular localizations. ν = 0.40, 0.48, 0.29, 0.17, and 0.10 for cytoplasm, cell periphery, punctate composite, nucleolus, and nucleus, respectively. The subcellular localization data were downloaded from http://www.umich.edu/~zhanglab/download/Wang_PLoSCB_Suppl/description.htm. Subcellular localizations containing >100 proteins and >30 interactions were shown. [file 1471-2148-10-358-S9.TIFF]
